# Activity-based protein profiling identifies alternating activation of enzymes involved in the bifidobacterium shunt pathway or mucin degradation in the gut microbiome response to soluble dietary fiber

**DOI:** 10.1038/s41522-022-00313-z

**Published:** 2022-07-20

**Authors:** Bryan J. Killinger, Christopher Whidbey, Natalie C. Sadler, Adrian J. DeLeon, Nathalie Munoz, Young-Mo Kim, Aaron T. Wright

**Affiliations:** 1 Biological Sciences Division, Pacific Northwest National Laboratory, Richland, WA 99352 USA; 2 The Gene and Linda Voiland School of Chemical Engineering and Bioengineering, Washington State University, Pullman, WA 99163 USA; 3 Chemistry Department, Seattle University, Seattle, WA 98122 USA

**Keywords:** Microbiome, Biological techniques

## Abstract

While deprivation of dietary fiber has been associated with adverse health outcomes, investigations concerning the effect of dietary fiber on the gut microbiome have been largely limited to compositional sequence-based analyses or utilize a defined microbiota not native to the host. To extend understanding of the microbiome’s functional response to dietary fiber deprivation beyond correlative evidence from sequence-based analyses, approaches capable of measuring functional enzymatic activity are needed. In this study, we use an activity-based protein profiling (ABPP) approach to identify sugar metabolizing and transport proteins in native mouse gut microbiomes that respond with differential activity to the deprivation or supplementation of the soluble dietary fibers inulin and pectin. We found that the microbiome of mice subjected to a high fiber diet high in soluble fiber had increased functional activity of multiple proteins, including glycoside hydrolases, polysaccharide lyases, and sugar transport proteins from diverse taxa. The results point to an increase in activity of the *Bifidobacterium* shunt metabolic pathway in the microbiome of mice fed high fiber diets. In those subjected to a low fiber diet, we identified a shift from the degradation of dietary fibers to that of gut mucins, in particular by the recently isolated taxon “*Musculibacterium intestinale”*, which experienced dramatic growth in response to fiber deprivation. When combined with metabolomics and shotgun metagenomics analyses, our findings provide a functional investigation of dietary fiber metabolism in the gut microbiome and demonstrates the power of a combined ABPP-multiomics approach for characterizing the response of the gut microbiome to perturbations.

## Introduction

In healthy mammalian hosts, microbes within the gastrointestinal (GI) tract form a mutually beneficial relationship with their host through the exchange of nutrients and metabolic products^1,2^. For example, carbohydrates that can be metabolized by the host are limited to mostly starch due to a lack of host-derived enzymes capable of metabolizing the wide array of polysaccharides commonly found in the diet^3^. These indigestible polysaccharides, termed dietary fiber, are commonly found in vegetables and fruit. In contrast to mammals, the metagenome of gut microbiota does encode for enzymes capable of degrading dietary fibers into nutrients that are tractable to the host and beneficial to the microbes^4^. Metabolic assimilation of these carbohydrates by gut microbes leads to the production of metabolites such as short-chain fatty acids (SCFAs) that can be absorbed by the host within the GI tract^5^. SCFAs can have substantial effects on the host by regulating gut barrier function, inflammation, and host metabolism^6–8^. In contrast to the benefits provided by increased fiber consumption, a low fiber diet has a considerably negative impact on host health. Increased susceptibility to pathogens, obesity, type 2 diabetes, cancer, and cardiovascular disease have all been associated with a low fiber diet^9–11^. A mechanistic understanding for these correlations has yet to be fully uncovered but is needed, because the literature suggests that these adverse health outcomes may largely be caused by the response of the gut microbiome^12,13^.

Studies on the effect of dietary fiber intake on the microbial composition of the gut has revealed bacteria capable of degrading dietary fiber tend to increase in abundance in humans when they are fed a plant-based diet^14^. A westernized low fiber diet reduces microbial diversity, while a diet rich in dietary fiber increases the capacity for production of SCFAs such as acetate, butyrate, and propionate. While sparse, there is evidence that low dietary fiber leads to selective pressure favoring gut microbes that do not rely on carbohydrates ingested by the host for energy^11^. Other sources of carbohydrates, such as the mucin glycans of the protective mucosal barrier lining the gut, may provide an alternative energy source for microbes encoding the appropriate enzymatic capabilities^15^. Mucin glycans vary in composition and configuration and contain sugars such as N-acetylgalactosamine, galactose, fucose, and N-acetylneuraminic acid (Neu5Ac). While there is a constant turnover of mucin glycans by gut bacteria in healthy individuals, certain conditions may optimize the environment for mucin-foraging bacteria to degrade the mucosal layer and provide access for pathogens to infect the underlying epithelial cells of the GI tract^11^. Specifically, deprivation of dietary fiber decreased the thickness of the gut mucosal barrier in gnotobiotic mice while increasing their susceptibility to the pathogenic bacteria *Citrobacter rodentium*. Corresponding transcriptional data indicated that the low fiber diet increased expression of microbial enzymes in the gut that likely release sugars from host glycans. This suggests that the decrease in observed mucosal barrier thickness was likely due to microbial degradation. However, this system utilized gnotobiotic mice colonized with a synthetic, 14-member microbial community based on genomic sequence availability and their relevance to human gut microbiota. As gut microbiota differ between mammalian hosts, direct measurement of the response of the native microbiota is needed may reveal microbial activity that cannot be observed in gnotobiotic models.

Identifying the molecular basis of interactions of dietary fiber and gut microbiota are needed to understand the impact on host health. Most studies of the gut microbiome rely on metagenomic or metatranscriptomic methods to predict community members and genes that are responsible for a given function. However, these studies can only identify a correlation between abundance and functional activity. To understand response of the gut microbiome to perturbations at a functional level, approaches capable of identifying and quantifying biochemical activity are needed. Activity-based protein profiling (ABPP) is one such approach^16^, which employs small molecules termed activity-based probes (ABPs) to enrich and measure functionally active enzymes that have an affinity for the ABP. Enriched proteins can then be identified by mass spectrometry, providing a direct measurement of protein function within complex systems such as the gut microbiome. The aim of this study was to characterize the gut microbiome response to dietary fiber at the functional level using ABPP coupled to microbial composition and metabolome analyses. This study addresses the hypothesis that a low fiber diet upregulates mucin foraging enzyme activity exposing the host to health consequences. We applied the sugar-derived activity-based probe GH2c-ABP^17^ to the GI content of mice fed diets either high or low in soluble dietary fiber to enrich microbial proteins with an affinity for the sugar moiety of the probe. We then analyzed the multi-omics and ABPP measurements and identified significant alterations to microbial protein activity between the high and low fiber groups.

## Results

### GH2c-ABP enriches carbohydrate active enzymes from the microbiome

**Fig. 1:**
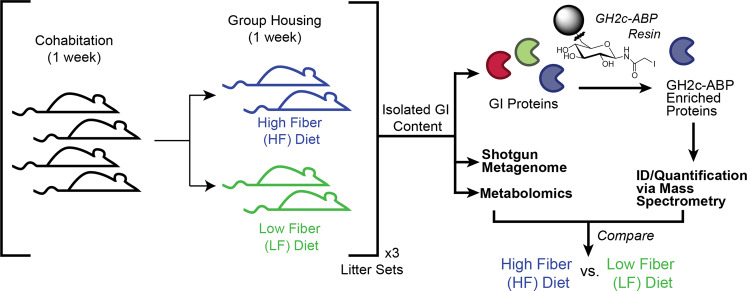
Experimental design for functional resolution of the microbiome response to altered fiber diets. Four littermates were cohabitated for 1 week before splitting into high fiber (HF) and low fiber (LF) littermate pairs. After 1 week, GI content was investigated with ABPP and multi-omics analyses. A GH2c-ABP linked resin was used to enrich proteins with metabolic or transport activities for sugar moieties from the GI proteome. Enriched proteins were then identified and quantified using mass spectrometry. The gut metagenome for each mouse was obtained via shotgun sequencing. Metabolites were analyzed via mass spectrometry. Omics measurements for the HF and LF groups were then compared.

As a model system, we established littermates (three sets of four mice each) that were provided with dietary pectin and inulin (high soluble fiber or HF; two littermates per set) or cellulose (low soluble fiber or LF; the other two littermates per set) (Fig. 1).

**Fig. 2:**
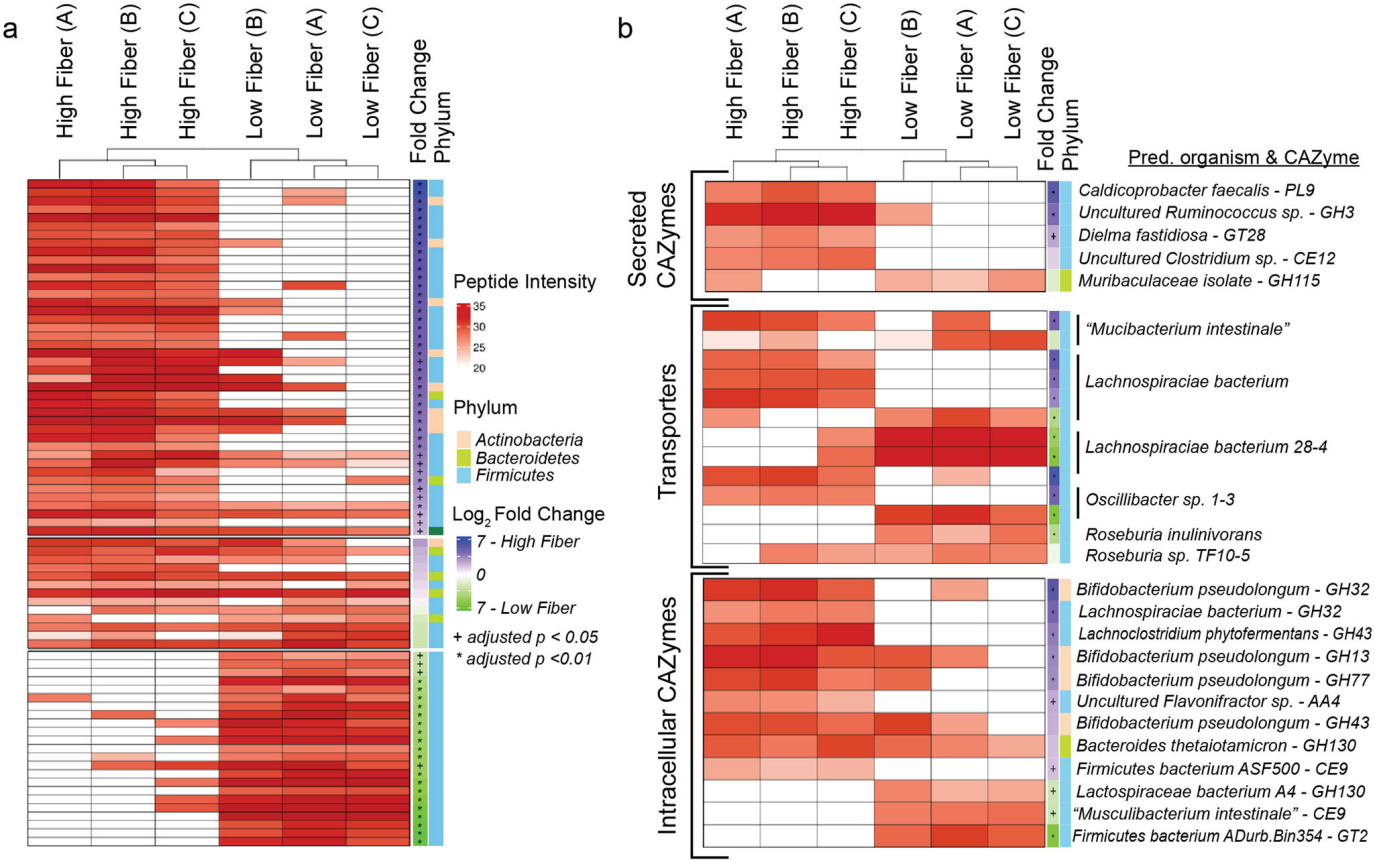
Distinct enzymes revealed by ABPP as active in high and low fiber conditions. Peptide intensities shown in red on the heatmap indicate the log_2_-transformed sum of unique peptide intensities for proteins that were targeted by the GH2c-ABP (rows) for each littermate sample group (columns). White cells indicate the protein was not detected in the specified littermate sample group. Littermate sample groups were clustered using Aitchison distance. A blue log_2_ fold change indicates enrichment in HF conditions while green indicates proteins enriched under LF conditions (directly right of heat map; * *q*-value < 0.05; + *q* value < 0.01). The predicted phyla that produced the corresponding protein is shown in the rightmost column (Tan: Actinobacteria; light green: Bacteroidetes; light blue: Firmicutes). **a** The 78 carbohydrate-related proteins identified by ABPP. **b** A subset of the 78 carbohydrate-related proteins consisting of putative CAZymes and transporters.

The GH2c-ABP (Fig. 1, top right) contains three distinct parts: an affinity group designed to bind to target enzymes (a sugar moiety), an enrichment group to enable isolation of active proteins (a bead conjugated to the probe using copper-catalyzed azide alkyne cycloaddition), and a reactive group to form a covalent bond with the target protein (iodoacetamide). Because of the comparably wide-ranging reactivity of iodoacetamides with nucleophilic residues, the GH2c-ABP was applied to broadly identify and measure the functional activity of sugar-binding proteins present in the GI content of the mice via LC-MS/MS measurements. This resulted in the identification of 295 proteins containing uniquely identified peptides. Statistical analysis with PECA^18^ resulted in the identification of 50 proteins enriched in the LF group and 107 proteins enriched in the HF group (*q* value < 0.05; all protein results use a *t* test with moderated t-statistic) while the remaining 138 had no significant difference between groups (*q* value ≥ 0.05). Of the total 295 identified proteins, we were able to annotate 78 via sequence alignments to the UniProt database (*E* value < 1E-9, see Supplementary Table 1)^19–21^ or by mapping their sequences to Cluster of Orthologous Groups orthologs with the eggNOG Mapper^22–25^. From these 78 proteins, 42 were significantly enriched under HF conditions (*q* value < 0.05), 23 enriched under LF conditions (*q* value < 0.05), and 13 did not reach statistical significance (*q* value ≥ 0.05). Proteins were selected for further analysis if a CAZy domain was confidently assigned (≥2 tools from dbCAN2). A selection of these proteins reveals enzymes produced by many distinct taxa with functions ranging from ABC transporters and isomerases to glycosidases and kinases (Fig. 2).

### A high fiber diet results in elevated activity of carbohydrate transporters from diverse taxa and excreted CAZymes

Given that secreted proteins play a key role in degradation of dietary fibers, we searched for secretion signals in all of our detected probe-targeted proteins using SignalP^26^. Under HF conditions, we detected increased activity for three carbohydrate-active enzymes (CAZymes) that had predicted secretion signals. One of these proteins has a PL9 polysaccharide lyase domain and aligned to a protein sequence from the *Firmicutes* taxa *Caldicoprobacter faecalis* (Fig. 2b). Characterized members of this family have demonstrated pectate lyase activity^27–29^. The other two CAZymes with predicted secretory signals were a glucosidase belonging to the GH3 family and a GT28-family glycosyltransferase (Fig. 2b). The majority of secreted proteins that were more active under HF conditions are annotated as solute binding protein (SBP) components of ABC transporters. Additionally, we found several organisms that produced distinct proteins under the different conditions (Fig. 2b).

### The bifidobacterial “bifid shunt” pathway and α-glucan metabolism are more active in a high fiber environment

**Fig. 3:**
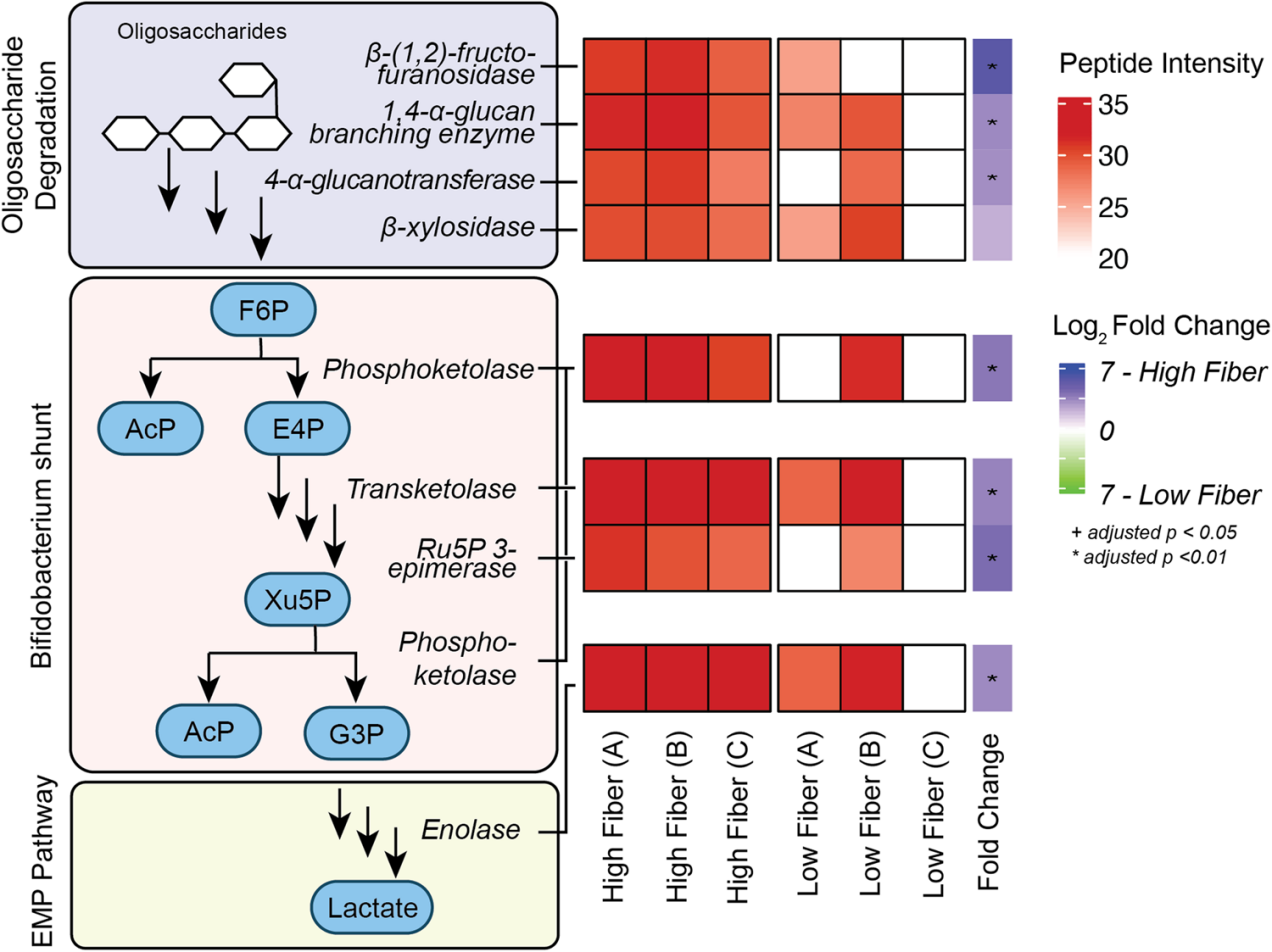
ABPP identifies Bifidobacterium enzymes involved in carbon catabolism with increased activity under HF conditions. Of the eight bifidobacterial enzymes, seven were significantly enriched (*q* value < 0.05) under HF conditions. Steps of the pathway are shown to the left and a heatmap of protein intensity is shown on the right. The log_2_-transformed sum of unique peptide intensities for proteins is shown in red, while white cells indicate the protein was not detected in that sample group. Imputed log_2_ fold change is shown on the right where blue indicates proteins enriched in HF conditions and green indicates proteins enriched under LF conditions.

We detected eight enzymes produced by *Bifidobacterium pseudolongum*, which is commonly found in the GI tract of mammalian and avian hosts^30,31^. A defining feature of bifidobacterial carbon metabolism is the presence of an alternative carbohydrate fermentation pathway known as the “bifid shunt”^32,33^. This pathway is defined by a key enzyme, fructose-6-phosphoketolase, and is theoretically capable of generating more moles of ATP per mole of glucose than the more common Embden–Meyerhof Parnas pathway. Among the eight *B. psuedolongum* enzymes we detected, seven were significantly more active in the HF group (*q* value < 0.05; *t* test with moderated t-statistic) and included the bifid shunt enzymes fructose-6-phosphate phosphoketolase, transketolase, ribulose phosphate 3-epimerase, and the downstream enolase (Fig. 3).

Relevant to the upstream degradation of inulin, we also detected a GH32-family β-fructofuranosidase as well as two proteins predicted to be involved in α-glucan metabolism: a GH13 1,4-glucan branching enzyme and a GH77 amylomaltase^34–36^. The substrate of these enzymes may be the provided dietary fiber or endogenous glycans such as bacterial glycogen. One predicted β-xylosidase was also identified with increased abundance under HF conditions, though it did not reach statistical significance.

### Low fiber conditions result in elevated activity of mucin glycan degradation by *“M. intestinale”*

**Fig. 4:**
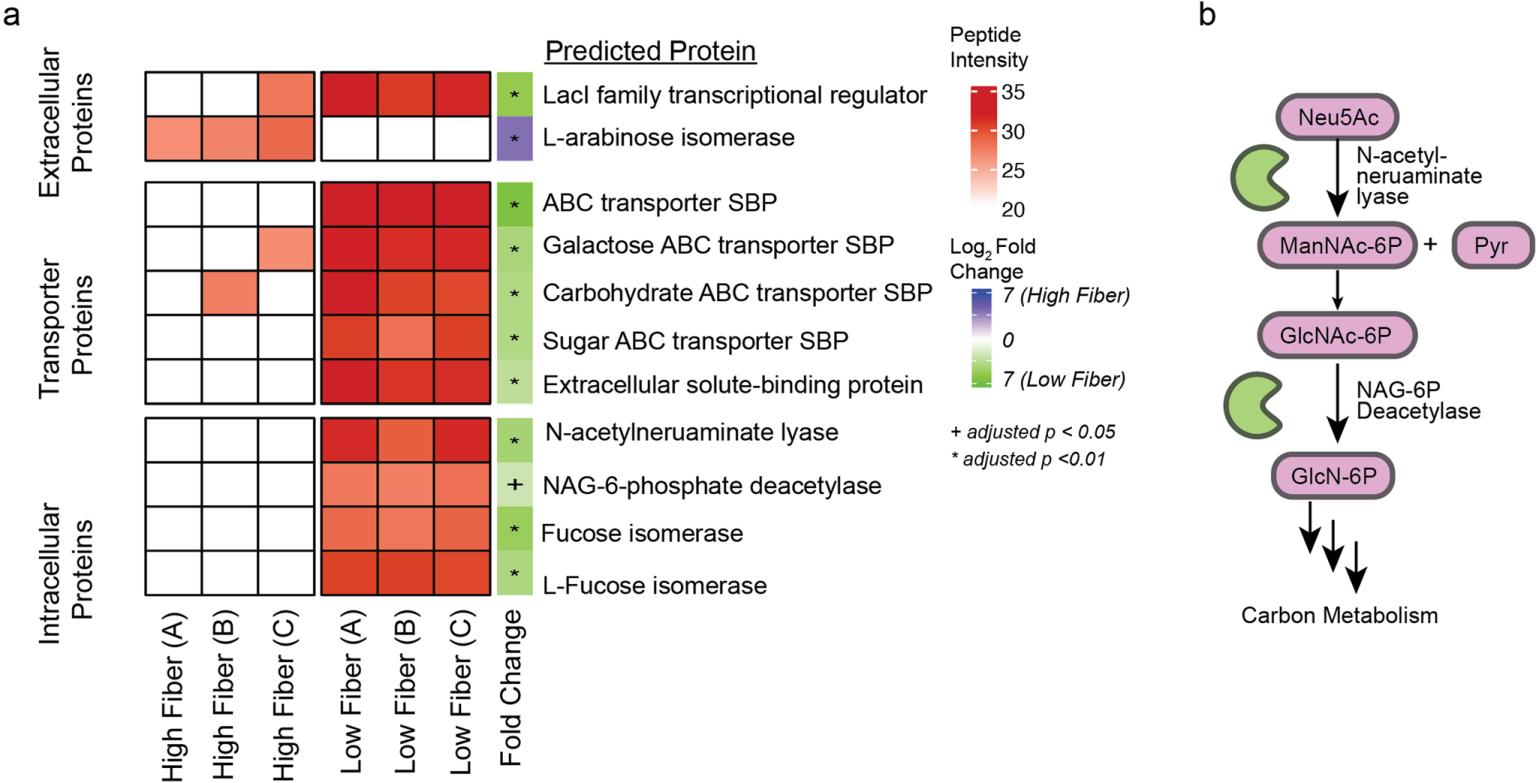
*“M. intestinale”* proteins involved in carbohydrate uptake and sialic acid (Neu5Ac) breakdown are more active under LF conditions. **a** 10 of 11 identified *“M. intestinale”* proteins were significantly enriched under LF conditions (*q* value < 0.05), while a predicted L-arabinose isomerase was only detected under HF conditions. The log_2_-transformed sum of unique peptide intensities for proteins is shown in red, while white cells indicate the protein was not detected in that sample group. Imputed log_2_ fold change is shown on the right (blue: enriched in HF conditions, green: enriched in LF conditions). **b** Two key enzymes in Neu5Ac degradation—an N-acetylneuraminate lyase and an N-acetylglucosamine-6P-deacetylase—were more active under LF conditions, indicating *“M. intestinale”* actively catabolizes Neu5Ac in the absence of soluble dietary fiber.

Of the 23 annotated proteins significantly enriched in the LF group, many were predicted to be carbohydrate ABC transporter SBPs from various microbes belonging to the Firmicutes phylum (Fig. 2b). The most commonly identified organism producing enriched proteins in the LF group was the recently described Bacterium 1XD8-92 or “*Musculibacterium intestinale*”^37^. This organism was isolated from the feces of a Lep^*ob*/*ob*^ or leptin-deficient C57BL/6J mouse and is genetically similar to *Eisenbergiella tayi*, a human-associated isolate assigned to the family *Lachnospiraceae*^38^. Of the 10 *“M. intestinale”* proteins with elevated activity in the LF group, five are carbohydrate ABC transporter SBPs and two are enzymes involved in the degradation of Neu5Ac (Fig. 4a).

Neu5Ac is a monosaccharide commonly found as a terminal residue of mucin glycans, which are essential components of the mucosal barrier lining the GI tract. We identified elevated activity in the LF group of the first key enzyme involved in degradation of Neu5Ac, N-acetylneruaminate lyase (NanA), which converts Neu5Ac to pyruvate and N-acetylmannosamine (Fig. 4b). N-acetylmannosamine can further be converted to fructose-6-phosphate by four enzymes, one of which, N-acetylglucosamine-6-phosphatase (NagA), was also enriched under LF conditions.

### Metagenomics supports ABPP by providing evidence of increased mucin glycan degradation and *“M. intestinale”* abundance in response to low dietary fiber

To support the ABPP analysis, we assigned a lowest-common ancestor to metagenomic reads using Kraken2^39^ and quantitatively compared estimated taxonomical abundances between the HF and LF groups using LEfSe^40^ (see Supplementary Table 2). Out of 19,062 taxa with kingdom to subspecies taxonomic ranks, we identified 12 taxa with significantly elevated abundance due to the HF diet and 12 with significantly elevated abundance due to the LF diet (LDA-score > 3.0, *P* value < 0.05). In the HF group, we observed an increase in the *Bacteroides* genera (5.13 LDA score, *P* value < 0.004), aligning with similar observations from previous microbiome studies^41–43^. Notably, in the LF group, we found *“M. intestinale”* to have a significantly elevated abundance (3.69 LDA score, *P* value < 0.004). In terms of relative abundance, an average of 1.11% of total bacterial reads were assigned to *“M. intestinale”* per sample in the LF group while only 0.06% were assigned to *“M. intestinale”* in the HF group. These observations align with our ABPP analysis and provides evidence of this organism’s ability to thrive under LF conditions.

We then sought to determine how dietary fiber impacted the functional capacity of the gut metagenome. Protein coding sequences from assembled metagenomes were annotated with Gene Ontology (GO) identifiers and statistically tested for enrichment of function between the HF and LF groups (see Supplementary Table 3). Out of 1332 GO functional identifiers, we identified 8 with differential abundance between dietary conditions (*q* value < 0.05; all *q*-values were determined using a Kruskal-Wallis sum-rank test followed by Wilcoxon rank-sum test)^44^. The LF diet led to a statistically significant increase in reads mapping to exo-alpha-sialidase activity [GO:0004308] (1.19 log_2_ fold increase; *q* value < 0. 0.0118), indicating an increase in the total metagenomic capacity for cleavage of terminal mucin sialic acid residues such as Neu5Ac^45^. In contrast, the HF diet led to statistically significant increase in reads mapping to GO identifiers for galactarate dehydratase activity [GO:0008867] (0.955 log_2_ fold increase; *q* value < 0.0232) and butyryl-CoA dehydrogenase activity [GO:0004085] (0.9355 log_2_ fold increase; *q* value < 0.0198). Notably, the GO identifiers corresponding to the ABPP targets NanA and NagA were not identified as being significantly enriched under LF conditions.

### Metabolomics data supports changes in enzymatic activities detected by ABPP

**Fig. 5:**
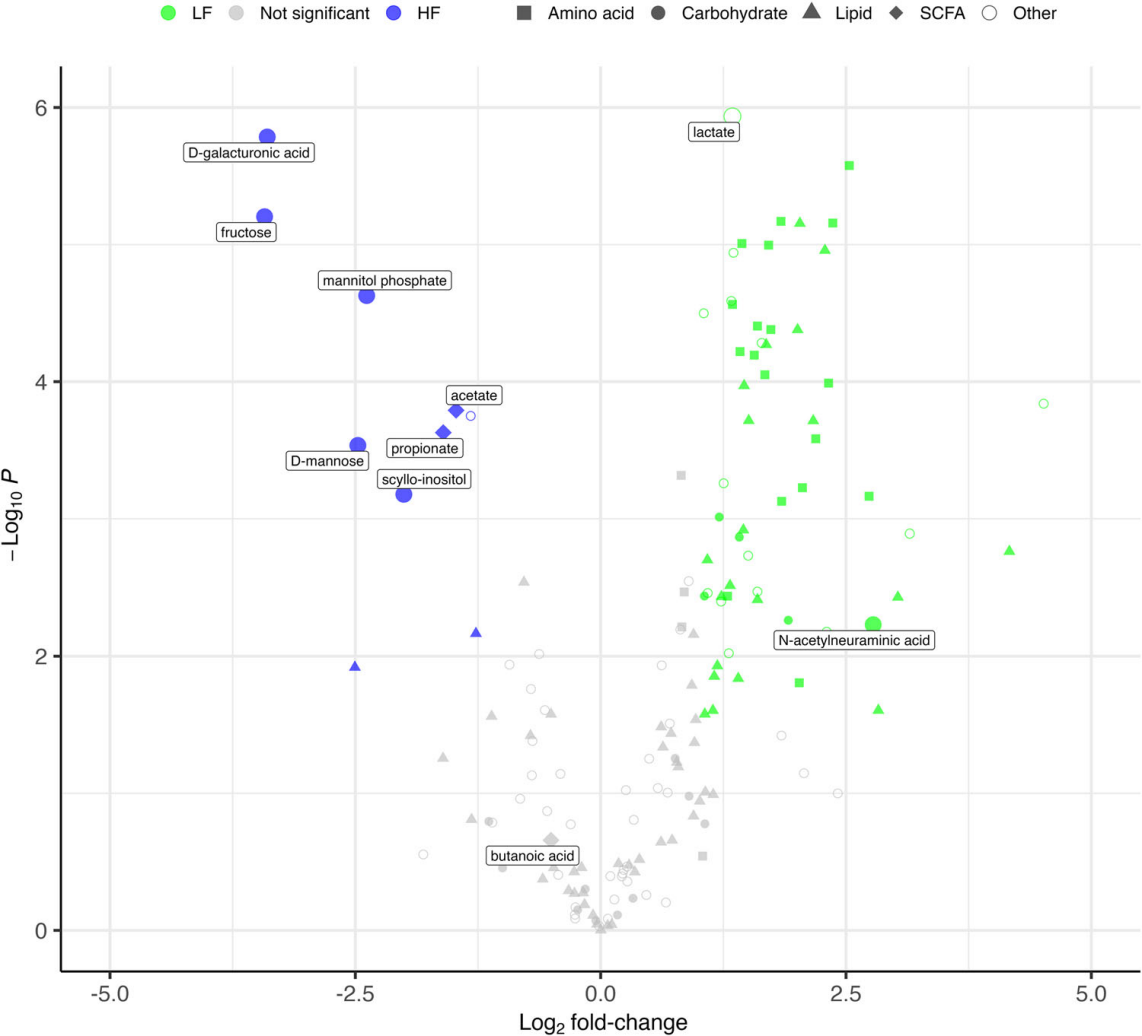
Differentially abundant metabolites in the GI tract in response to inulin and pectin. Volcano plot of detected metabolites. Significantly differentially abundant metabolites (*q* value < 0.05, |log_2_ fold-change| > 1.0) are labeled and colored green or blue, while metabolites with no significant difference between groups are colored gray. A positive (green nodes) or negative (blue nodes) log_2_ fold-change indicates a relatively higher abundance in the LF or HF group respectively. Node shapes indicate metabolite class while nodes indicating metabolites discussed in the text are enlarged and labeled.

In addition to ABPP and metagenomic sequencing analysis, we investigated how the gut metabolome changed in response to the availability of dietary fibers (Fig. 5, see Supplementary Table 4). We observed that the HF diet resulted in a relative increase in abundance of the dietary fiber components galacturonic acid and fructose (Fig. 5a). The HF diet also resulted in a significant increased abundance of the microbial-derived SCFAs acetate and propionate. Increased amounts of butyrate did not reach statistical significance. Other metabolites such as mannose, mannitol-phosphate, and scyllo-inositol were also more abundant under HF conditions. When compared to the HF diet, the LF diet resulted in a significant relative increase of all detected proteogenic amino acids and many lipids. Notably, the gut mucin sugar Neu5Ac was significantly more abundant in the LF group (Fig. 5).

## Discussion

A major step toward applying gut microbiome research to improve health outcomes is the ability to identify causal relationships between biochemical function and specific taxa and enzymes. As such, it is important to apply new techniques that couple protein identification to a measurement of activity. ABPP is well suited to this challenge. In this study we performed a multi-omics analysis incorporating ABPP to investigate the functional response of the mouse gut microbiome to high or low dietary fiber conditions at the molecular level.

Organisms present in the gut microbiome interact with each other in several ways. One type of interaction is cross-feeding, in which the products of one organism’s metabolic activity may serve as a carbon or energy source for another^46^. In some cases, the product may be a “public good” where both the metabolite producer and other community members are capable of further metabolizing the product. This has been previously demonstrated for *Bifidobacteria* strains and *Bacteroides* strains grown on dietary polysaccharides in vitro^47,48^. Using ABPP, we found that most secreted proteins in vivo were carbohydrate ABC transporter SBPs from diverse taxa, while only a few secreted proteins were enzymes involved in fiber degradation. This suggests that when dietary fiber is provided, the identified taxa producing these transporters are more active in assimilating the oligo- and monosaccharide components of soluble dietary fibers liberated by secreted CAZymes such as the PL9-containing protein described above. This suggests that a “public good” type of cross-feeding may be occurring using the soluble fiber as a substrate. Intriguingly, we also found different proteins from the same taxon to be active under different conditions. This may be due to strain-level differences, or it may be that the taxon persists under both conditions but switch active proteins depending on substrate availability. Incorporating metatranscriptomics and global metaproteomics would help elucidate what level or levels of regulation control that switch.

In the high fiber group, we observed elevated activity of enzymes produced by *B. pseudolongum* involved in the degradation of fructo-oligosaccharides (FOS) via the “bifid shunt” pathway. Downstream of FOS degradation, our results show that *B. pseudolongum* produces active bifid shunt enzymes in vivo, including the fructose-6-phosphate phosphoketolase central to this pathway. To our knowledge, this is the first evidence of in vivo bifid shunt pathway activity at the enzyme level in a mammalian GI tract. These data connect to another type of cross-feeding where the waste products of the bifid shunt (acetate and lactate) are utilized by other organisms as a carbon source. Both acetate and lactate were detected in our metabolomics analysis. While acetate was present at higher levels under HF conditions, lactate was more abundant under LF conditions. The exact ratio of lactate to acetate produced by bifidobacterial fermentation is reported to be dependent on strain, carbohydrate source, and environment^49^. In our system, lactate may serve as a cross-feeding metabolite under HF conditions that is further degraded by other gut community members. This would agree with the observation that propionate, a product of cross-feeding, is elevated under HF conditions as well^50,51^. Alternatively, lactate could be produced as an end product under LF conditions, leading to a higher relative abundance. Absolute quantification of key metabolites could serve as a way to address this question.

Previous work has demonstrated that the absence of dietary fiber results in degradation of the mucosal barrier and leads to an increased susceptibility to pathogenic infection^15,52^. In our LF group, we found an increase in activity of specific enzymes predominantly produced by the recently isolated organism *“M. intestinale”*. Two of these enzymes are involved in the assimilation of the mucin-derived monosaccharide Neu5Ac, providing functional enzymatic evidence for degradation of the mucosal layer under LF conditions. Shotgun metagenomics and metabolomics supported the ABPP analysis and revealed that dietary fiber deprivation results in an increase of *“M. intestinale”* abundance, increased total metagenomic capacity for exo-alpha-sialidase activity, and increased abundance of free Neu5Ac. Thus, each omics analysis yielded complementary information regarding microbial Neu5Ac degradation and assimilation in response to dietary fiber deprivation.

Our functional study does have some limitations. An increased number of biological replicates would increase the power of the findings, and larger sample sizes would provide more material for proteomics analysis. Our studies will extend to clinical cohorts of human stool, which will expand sample number and size. ABPP, while powerful, still requires a search database built from available genomes. We have determined that characterizing the taxa to build the database is the best current approach, but as informatics analyses grow and strengthen in microbiome studies we anticipate results will further improve. As performed, proteins deriving from an organism not included in the database or lacking an available genome were not detected. As more species- and strain-resolved genomes become available, this may help identify additional enzymes that are not present in current databases.

Ultimately, our results demonstrate the capability of an ABPP-based multi-omics approach to characterize the microbiome’s response to perturbations, and we anticipate this approach will provide further functional insights in future microbiome studies.

## Methods

### Experimental design

To investigate the effect of dietary fiber on the microbiome, we employed metagenomic, metabolomic, and ABPP analyses to the GI content of mice fed diets that varied in fiber content. Three sets of four female C57BL/6J littermates age 6–8 weeks were purchased from Jackson Laboratories and housed with a 12-h light/12 hour dark light cycle. High fiber rodent chow (HF, TD.140006) and low fiber chow (LF, TD.140004)^11^ was purchased from Envigo (Indianapolis, IN, USA). Chow and water were provided ad libitum. Mice were co-housed with littermates and provided with standard diet for 7 days prior to the onset of treatment (PMI 5001) purchased from Animal Specialties, Inc. After this time, each litter was divided into two groups for dietary treatment (2 mice per litter set). One group from each set of littermates (*n* = 6 total) were given HF chow containing inulin (75 g/kg) and pectin (75 g/kg), while the other groups (*n* = 6 total) were given LF chow analogous to the HF chow except increased maltodextrin (+20%, +19.8 g/kg), cornstarch (+18%, +55.2 g/kg), and cellulose (+300%, +75 g/kg) were substituted for pectin and inulin. Pectin was derived from citrus, and contained >74% galacturonic acid. The degree of esterification was >65% and the degree of methylation was >6.7%. Inulin was derived from chicory root and had a reported degree of polymerization of 10 (Cargill Oliggo-Fiber). Mice were maintained on this diet for 7 days, during which time weight was monitored. After 7 days, mice were euthanized and GI tracts were removed, stored in phosphate-buffered saline (PBS) and frozen at −80 ^o^C until further processing. Each analysis incorporated a hierarchical experimental design identifying dietary effects between the HF and LF groups while accounting for littermate groups. All statistical tests are two-sided with further details in their respective subsection.

### Ethics declaration

This study was approved by the Institutional Animal Care and Use Committee at Pacific Northwest National Laboratory (Protocol 2015-14).

### Isolation of small molecule metabolites and metabolomic measurements

GI tracts were thawed and GI content was isolated into sterile tubes and weighed. Approximately one quarter of the GI content was placed into a clean Eppendorf tube and weighed. Metabolites were extracted and further processed for polar and volatile metabolomics analysis^53,54^. For the global and non-volatile metabolomics analysis, the GI content was extracted using the MPLEx protocol extraction. Briefly, 400 µl of a chloroform: methanol (2:1) solution were added to ~100 mg of sample and kept on ice for 10 min. Samples were vortexed for 30 s and centrifuged at 17,000 *g* at 4 °C for 5 min. Upper and lower phases were transferred to a glass vial and dried in a speed-vac concentrator. Dried extracts were derivatized in a two-step reaction (55). To protect carbonyl groups and reduce the number of tautomeric isomers, 20 µl of methoxyamine in pyridine (30 mg/ml) was added to each dried extract, followed by vortexing for 30 s and incubation at 37 °C with shaking (1000 r.p.m.) for 90 min. To derivatize hydroxyl-, amino-, carboxyl- and thiol- groups to trimethylsilylated forms, 80 µl of N-methyl-N-(trimethylsilyl)trifluoroacetamide with 1% trimethylchlorosilane were then added to each vial, followed by vortexing for 10 s and incubation at 37 °C with shaking (1000 r.p.m.) for 30 min. Samples were run in an Agilent GC 7890A using a HP-5MS column (30 m × 0.25 mm × 0.25 μm; Agilent Technologies) coupled with a single quadrupole MSD 5975C (Agilent Technologies). One microliter of sample was injected into a splitless port at constant temperature of 250 °C. The GC temperature gradient started at 60 °C, with a hold of temperature for 1 min after injection, followed by increase to 325 °C at a rate of 10 °C/min and a 5-minute hold at this temperature. A fatty acid methyl ester standard mix (C8-28) (Sigma-Aldrich) was analyzed in parallel as standard for retention time calibration. GC-MS raw data files were processed using the Metabolite Detector software. Retention indices of detected metabolites were calculated based on the analysis of a FAMEs mixture, followed by their chromatographic alignment across all analyses after deconvolution. Metabolites were initially identified by matching experimental spectra to a PNNL augmented version of Agilent GC-MS metabolomics Library, containing spectra and validated retention indices for over 850 metabolites. Then, the unknown peaks were additionally matched with the NIST17/Wiley11 GC-MS library. All metabolite identifications and quantification ions were validated and confirmed to reduce deconvolution errors during automated data-processing and to eliminate false identifications.

For the volatile metabolomics analysis, the GI content was mixed with methanol in a 1:1.5 ratio. The slurry was centrifuged for 10 min at 4 °C and 60 µl of the upper part of the solution were transferred to glass vials equipped with inserts for direct GC-MS analysis without chemical derivatization. Samples were run in the same Agilent GC-MS instrument mentioned above but equipped with a HP-FFAP column (30 m × 0.250 mm × 0.250 μm; Agilent Technologies) (54). One microliter of sample was injected into a splitless port at a constant temperature of 240 °C. The GC temperature gradient started at 50 °C, with a hold of temperature for 1 min after injection, followed by increase to 240 °C at a rate of 20 °C/min and a 3.5-min hold at this temperature. Two technical replicates were injected of each sample. A fatty acid methyl ester standard mix (C8-28) (Sigma-Aldrich) was analyzed in parallel as standard for retention time calibration. Processing of GC-MS raw data files and identification of metabolites was done as detailed in the previous section.

### Isolation and sequencing of DNA

Approximately one-quarter of the GI content sample was aliquoted and utilized for metagenomic sequencing. DNA was isolated using the DNeasy PowerSoil Kit (Qiagen) per manufacturer’s instructions. The library preparations and sequencing were conducted by GENEWIZ, Inc (Whole Metagenome Sequencing service; South Plainfield, NJ). DNA was sequenced on an Illumina HiSeq in the 2x150bp configuration.

### Activity-based protein profiling

#### Preparation of gut microbiome protein samples

The remaining approximate one-half of GI content was utilized for ABPP. Because of low biomass, samples from the two littermate mice of the same treatment groups were pooled. Pooled GI content was transferred to a 50 mL conical tube and resuspended in 10 mL of PBS. The sample was centrifuged at 700 *g* for 5 min to clear large debris. This supernatant was transferred to a new 50 mL conical tube and centrifuged at 7000 *g* for 5 min to collect microbial cells. The pellet from this centrifugation was resuspended in 1 mL of acetate-buffered saline (50 mM acetate, 150 mM NaCl, pH = 5.0) and transferred to a 1.7 mL Eppendorf tube. This sample was centrifuged again at 7000 *g*, and the pellet was washed two additional times via resuspension in 1 mL of acetate-buffered saline and centrifugation. The pellet was then resuspended in 600 μL of acetate-buffered saline with EDTA-free protease inhibitor cocktail (Roche). Approximately 100 μL of 0.1 mm silica beads were added and cells were lysed via 4 rounds of bead beating (Bullet Blender). The tubes were centrifuged at 7000 *g* for 15 min to remove beads and unlysed cells. The supernatant was transferred to a new Eppendorf tube and the concentration of protein was determined via BCA assay (Thermo). Protein volumes were adjusted to equal the lowest concentration (0.134 mg/mL).

#### Preparation of probe-conjugated resin

To minimize enrichment of off-target proteins, we covalently attached the GH2c-ABP to resin prior to labeling. To this end, 300 μL of alkyne-agarose resin (six samples at 50 μL resin per sample; Click Chemistry Tools) was aliquoted into Eppendorf tubes, centrifuged (1 min at 600 *g*) to collect resin. In tandem, a second aliquot of resin was prepared as a “no probe” control. The supernatant was removed, resin was washed with 1 mL of PBS, and centrifuged again. Three total washes were performed, and resin was resuspended in 500 μL of PBS. The amount of probe was calculated based on the desired concentration of probe in the final reaction (100 μM), assuming 100% conjugation. Probe was conjugated to the resin using copper-catalyzed azide-alkyne cycloaddition via the addition of 500 μM GH2c, 20 mM CuSO_4_, 10 mM THPTA, 25 mM sodium ascorbate. In tandem, a second aliquot of resin was prepared as a “no-probe” control. Resin was processed in the exact same way, except GH2c was replaced with an equal volume of vehicle only (DMSO). The tubes were wrapped in foil and attached to a vortex shaker and mixed for 90 min at room temperature. The resin was washed three times in PBS as described above prior and aliquoted equally into one 1.7 mL Eppendorf tube per sample.

#### Enrichment of proteins targeted with the GH2c-ABP

The aliquoted, GH2c-conjugated resin or the no probe control resin was resuspended in 200 μL of sample and incubated rotating at 37 °C for 90 min. The resin was then collected via centrifugation (1 min at 600 *g*) and resuspended in 1 mL of 4% sodium dodecyl-sulfate in PBS. The tubes were then incubated rotating at 37 °C for one hour. After this point, ABPP samples were washed 3× with PBS, digested with trypsin in PBS, and the buffer was removed by evaporation^55^. Peptides were resuspended in 25 µL ammonium bicarbonate (25 mM, pH 8) for subsequent MS analysis.

#### LC-MS/MS proteomics analysis

A Waters nano-Acquity M-Class dual pumping UPLC system (Milford, MA) was configured for on-line trapping of a 5 µL injection at 3 µL/min with reverse-flow elution onto the analytical column at 300 nL/min. Columns were packed in-house using 360 µm o.d. fused silica (Polymicro Technologies Inc., Phoenix, AZ) with 5-mm Kasil frits for media retention and contained Jupiter C18 media (Phenomenex, Torrence, CA) in 5 µm particle size for the trapping column (150 µm i.d. × 4 cm long) and 3 µm particle size for the analytical column (75 µm i.d. × 70 cm long). Mobile phases consisted of (A) 0.1% formic acid in water and (B) 0.1% formic acid in acetonitrile with the following gradient profile (min, %B): 0, 1; 8, 1; 10, 8; 28, 12; 83, 30; 105, 45; 108, 95; 118, 95; 122, 50; 124, 95; 126, 1; 128, 50; 130, 50; 132, 1; 152, 1.

MS analysis was performed using a Q-Exactive Plus mass spectrometer (Thermo Scientific, San Jose, CA) outfitted with a home-made nano-electrospray ionization interface. Electrospray emitters were prepared using 150 μm o.d. × 20 μm i.d. chemically etched fused silica^56^. The ion transfer tube temperature and spray voltage were 300 °C and 2.2 kV, respectively. Data were collected for 100 min following a 20 min delay from sample injection. FT-MS spectra were acquired from 300 to 1800 m/z at a resolution of 35k (AGC target 3e6) and while the top 12 FT-HCD-MS/MS spectra were acquired in data dependent mode with an isolation window of 2.0 *m*/*z* and at a resolution of 17.5k (AGC target 1e5) using a normalized collision energy of 30 and a 30 s exclusion time.

### Activity-based protein profiling data analysis

#### Prediction and annotation of protein-coding sequences from metagenome sequencing

Predicted open-reading frames from assembled metagenomes were used for the generation of a searchable protein-sequence database for matching theoretical spectra to experimental proteomics data. To do this, we began with the cleaning of metagenomic sequencing reads from samples of GI content matching those used in our proteomics experiments. First, we removed low-quality bases and adapters from our metagenome paired-end reads with Trim Galore^57^ (Trim Galore v0.6.4, q-cutoff of 15 as defined by the software package). Reads for each sample were then assembled using the metagenomic assembler metaSpades^58^ (SPAdes v3.13.1 [metaSPAdes mode], paired-end reads, all other options as default). Open-reading frames were predicted from the assembled metagenomes with Prodigal^59^ (Prodigal v2.6.3, metagenome option, all other options as default). Predicted open-reading frames were then aligned against the combined UniProt^19^ TrEMBL and Swiss-Prot databases (release 2021_03) using the Diamond^60^ alignment software for functional and taxonomical annotation (Diamond v0.9.26, one high-scoring pair per sequence).

#### Peptide-spectrum matching of LC-MS/MS data

Regardless of alignment results, all predicted open-reading frames, in addition to a common contaminant sequence and all proteins in the canonical mouse proteome available from UniProt (release 2019_09), were then concatenated to generate a single FASTA file. Sequence duplicates were removed and sequences with duplicated names were renamed using the seqkit^61^ program (seqkit v0.11.0, sequence duplicate removal: seqkit rmdup; renaming of sequences: seqkit rename). This FASTA was then used in an initial MS-GF + ^62^ search to identify experimental tryptic peptides (MS-GF + v2019.07.03, default options). A second fasta file containing all full protein sequences encoding for any tryptic peptide from the initial search identified with a SpecEValue < 1e-8 was then generated. A second MS-GF + search was then performed with the reduced FASTA file while maintaining the same parameters as the initial search. In a similar fashion to other iterative approaches^63^, peptides detected with an FDR < 5% were selected for statistical quantitative comparison.

#### Peptide quantification and statistical comparison of probe-targeted proteins

Statistical comparison of proteomics data was performed on MS-GF + identified peptides with quantification obtained from the log_2_-transformated peak area intensity calculated by MASIC^64^ (MASIC v3.0.7235, default options). After combining MS-GF + and MASIC results, peptides that were not observed in all biological replicates for at least one of the comparative treatments or were identified as a known contaminant were removed from the analysis. Remaining missing peptide intensities were imputed using a left-censored stochastic minimal value approach from the imputeLCMD R package^65^ (R v3.6.1, imputeLCMD v2.0). The PECA^18^ R package (PECA v1.24.0, test: modified t-test, type: median) was then used for statistical comparison of peptide peak area intensities to obtain *P* values of probe-targeted proteins through comparison of probe and no-probe control samples. Probe-targeted proteins in either diet condition were then isolated for further analysis. Peptides from probe targets matching to multiple proteins were then removed so that only unique peptides matching to a single protein remained. PECA was then used to compare the enrichment of probe-targeted proteins between the high and low fiber diet conditions. Multiple hypothesis correction was then performed to determine probe targets with differential enrichment between the different dietary conditions (*q* value < 0.05, average log_2_ fold-change > 2.0; *q* value determined by *t* test with moderated t-statistic within PECA). Fold-changes for each protein were calculated by averaging their constituent peptide-level log_2_ fold-changes. Proteins were then matched to their aligned UniProt identifiers and annotated by sequential requests to the UniProt database via the UniProt API. Proteins for further analysis were selected as described in the text. Heatmaps were generated using the R packages ComplexHeatmap (PMID: 27207943) and robComposition^66^.

### Metagenomic taxonomy analysis

Kraken2 was used to calculate taxonomical abundances prior to differential abundance testing. To prepare a Kraken2 database that contained organisms likely present in our samples, protein coding sequences that aligned with 100% sequence similarity to sequences in the Uniprot database were mapped to their corresponding lineage identifiers via Uniprot. These lineage identifiers were then used to search for bacterial genomic assemblies on NCBI (accessed 7-14-2021), which were subsequently downloaded and used to prepare a Kraken2 database. After taxonomical assignments of metagenomic reads by Kraken2, LEfSe^40^ (LEfSe v1.0.0) was then used to detect differentially-abundant features between dietary conditions from Kraken2 output (*P* value < 0.05).

### Metabolomics data analysis

Intensities of identified metabolites were log_2_-transformed. Missing values were imputed using a left-censored stochastic minimal value approach from the R imputeLCMD package, similar to our proteomics analysis. Metabolites were then statistically tested for differential intensities between dietary conditions using multiple linear regression with the “lm” function available in R. Reported *P* values for each metabolite were corrected using the Benjamini-Hochberg multiple hypothesis correction. Significantly changing metabolites were determined as those with a *q*-value < 0.05 (*t* test).

### Gene Ontology functional analysis

Protein coding sequences that aligned to proteins in the Uniprot database with greater than 90% similarity were selected for functional analysis after the removal of human and mouse proteins. The corresponding Uniprot identifiers were externally cross-referenced to Gene Ontology (GO) functional identifiers on the Uniprot website. After filtering for functional identifiers with at least 100 observations across all conditions, the counts of each identifier were summed for each sample. A ﻿negative binomial generalized log-linear model from the R package edgeR^67^ (edgeR v3.26.8) was then used to statistically test for differential enrichment of GO functional identifiers (*q* value < 0.05; likelihood ratio test).

## Supplementary information

Supplementary Figure 1

Supplementary Tables

## Data Availability

The datasets supporting the conclusions of this article are available in various repositories. The mass spectrometry proteomics data have been deposited to the ProteomeXchange Consortium via the PRIDE^68^ partner repository and are available with the dataset identifiers PXD018644 and 10.6019/PXD018644. Metagenome sequences are available through EBI (Project PRJEB37383). Metabolomics data have been deposited to the EMBL-EBI MetaboLights database (DOI: 10.1093/nar/gkz1019, PMID:31691833) with the identifier MTBLS4876. The complete dataset can be accessed here https://www.ebi.ac.uk/metabolights/MTBLS4876. Analysis scripts can be found at https://github.com/brykpnl/microbiome_fiber.
