# Supplementary material for: Activity-based protein profiling identifies alternating activation of enzymes involved in the bifidobacterium shunt pathway or mucin degradation in the gut microbiome response to soluble dietary fiber
Source: NPJ Biofilms Microbiomes. 2022 Jul 20;8:60. doi: 10.1038/s41522-022-00313-z (PMC9300575; doi:10.1038/s41522-022-00313-z)
Supplement: Supplementary file 1 — Supplementary Figure 1 [file 41522_2022_313_MOESM1_ESM.pdf]

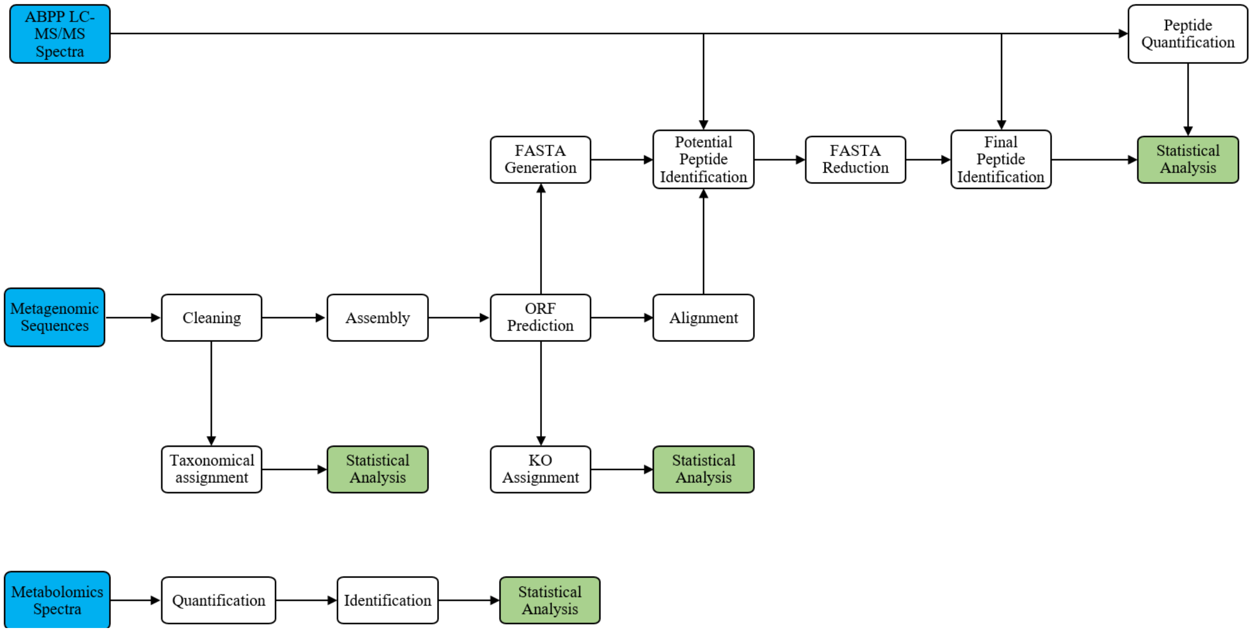

Supplementary Figure 1. Flowchart of analyses performed on multi-omics data. Statistical analyses were performed for each omics dataset to determine differential measurements observed between high fiber and low fiber conditions (see Methods section for details).
